# Self‐Regulated Moderate Intensity Habitual Exercise and Next‐Day Resting Metabolic Rate in Male Endurance Athletes: Implications for Athlete Testing

**DOI:** 10.1002/ejsc.70011

**Published:** 2025-07-10

**Authors:** Jack Eoin Rua O’Neill, Riona Joyce, Niamh Mc Loughlin, Jennifer Robinson, Ciara Mc Phillips, Barry O’Connell, Katy Horner

**Affiliations:** ^1^ Institute for Sport and Health and School of Public Health Physiotherapy & Sport Science University College Dublin Dublin Ireland; ^2^ School of Public Health, Physiotherapy & Sport Science University College Dublin Dublin Ireland

**Keywords:** endurance, metabolism, nutrition, physiology

## Abstract

Best practice guidelines for resting metabolic rate (RMR) testing are to avoid moderate to vigorous physical activity for 12–48 h beforehand, the upper limits of which can be difficult for athletes to adhere to. We investigated the effect of self‐regulated moderate intensity exercise in a free‐living setting on next‐day RMR in endurance athletes. Thirteen male endurance athletes participated, alternating between rest and exercise conditions in randomised order. For the exercise condition, participants were instructed to complete a habitual moderate intensity training session (rated 3–4 on the CR‐10 rate of perceived exertion scale) on the day before RMR measurement. Recovery markers (blood urea and subjective wellness) were assessed, and intra‐individual variation in RMR was explored. Mean (SD) habitual exercise session duration was 111 ± 71 min, heart rate was 128 ± 16 bpm and RPE score was 3.3 ± 0.5. Next‐day RMR did not statistically differ between exercise and rest conditions (1979 ± 289 vs. 1958 ± 251 kcal/day, mean Δ = 21 ± 227 kcal/day [95% CI = −116 to 158 kcal/day], *p* = 0.74, intra‐individual inter‐day CV 3.8% ± 3.4%). There were no significant correlations between changes in RMR and recovery markers. The findings suggest habitual moderate intensity exercise may be feasible on the day prior to RMR testing in male endurance athletes. Further investigation to perform equivalence testing between conditions is warranted.

## Summary

- Adhering to the upper limit of existing guidelines for RMR testing (up to 48 h of abstention from moderate to vigorous activity) can be challenging for athletes with frequent or daily training.
- This study examined the effect of self‐regulated, moderate intensity habitual exercise in male endurance athletes on RMR and associations with markers of recovery.
- RMR did not differ statistically between exercise and rest conditions in male endurance athletes. Larger studies, including female participants, are needed to inform consideration of changes to current guidelines regarding exercise avoidance.

## 1 Introduction

Resting metabolic rate (RMR) refers to the amount of energy expended by an individual at rest, accounting for as much as 60%–75% of total daily energy expenditure in the general population (Poehlman 1989). Measuring RMR assists in estimating energy requirements in athletes, and guiding nutritional and training interventions to support performance and recovery (Thomas et al. 2016). In the laboratory, the measurement of RMR is essential in studies assessing energy expenditure in athletes. This includes research utilising RMR ratio as an indicator of low energy availability (Staal et al. 2018; De Souza et al. 2007; Sterringer and Larson‐Meyer 2022), where the accurate measurement of RMR is paramount.

Current best practice guidelines for RMR measurement recommend a period of abstention from moderate to vigorous physical activity ranging from 12 to 48 h prior to measurement (Fullmer et al. 2015). This recommendation was assigned a ‘consensus’ grade based primarily on expert opinion rather than robust research evidence (Fullmer et al. 2015). Notably, the guideline authors highlighted the lack of clarity around how various physical activities (with differing intensities and durations) affect RMR and emphasise the need for further investigation, specifically pointing out that factors such as training status may play a role (Fullmer et al. 2015). While these guidelines aim to mitigate any acute impact of exercise on RMR, adhering to the upper limits of a 12–48 h abstention period from exercise can disrupt an athlete’s training regime. This creates a conflict between preserving strict measurement protocols and meeting the demands of regular training schedules.

Existing research demonstrates that high‐intensity aerobic exercise elevates RMR for up to 24 h (Treuth et al. 1996; Maehlum et al. 1986; Hunter et al. 2006) with some studies reporting increases for up to 48 h (Jamurtas et al. 2004). Protocols involved cycle ergometer intervals at 100% of VO_2_ max (Treuth et al. 1996) or sustained aerobic efforts at 70%–80% of VO_2_ max (Maehlum et al. 1986; Hunter et al. 2006; Jamurtas et al. 2004). In contrast, moderate intensity aerobic exercise has been shown to have no statistically significant effect on next‐day RMR (Kuikman et al. 2024). Recently, Kuikman and colleagues investigated the effects of acute dietary and exercise manipulations on next‐day RMR in male and female endurance‐trained athletes (Kuikman et al. 2024). The study incorporated five distinct pretest conditions, each varying in levels of energy availability and the inclusion or exclusion of pretest day exercise (Kuikman et al. 2024). In the exercise conditions, participants completed two cycling sessions, one in the morning and one in the afternoon at 55% and 65% of VO_2_max, respectively, resulting in a cumulative exercise energy expenditure on the day prior to RMR testing of 30 kcal per kg FFM (Kuikman et al. 2024). No statistically significant impact of dietary or exercise manipulations on next‐day RMR were observed (Kuikman et al. 2024).

In these previous studies, the training status of the participants must be considered as this could impact the response. For example, there is evidence of a reduced impact of resistance exercise on the next‐day RMR in participants who habitually perform resistance exercise (minimum 2 years training) compared to untrained individuals, which corresponded with markers of exercise recovery (Dolezal et al. 2000). In most of the previous studies investigating the effect of high‐intensity aerobic exercise on RMR, the physical activity status of the participants was not mentioned (Treuth et al. 1996; Jamurtas et al. 2004) or participants were classified as not participating in competitive sports (Maehlum et al. 1986). In the study in which elevated RMR was observed for up to 19 h post‐exercise, participants had been exercising for 8 weeks at the time of the RMR measurement; however, they were classified as sedentary prior to enrolment (Hunter et al. 2006). In contrast, in the recent study by Kuikman and colleagues showing no statistically significant effect of moderate intensity exercise on next‐day RMR (Kuikman et al. 2024), participants were endurance‐trained athletes of Tier 2 *(‘Trained/Developmental’)* to Tier 3 *(‘Highly Trained/National Level’)* calibre (McKay et al. 2022). Therefore, current training status of participants and the modality in which the pretest RMR exercise is performed may impact the response and must be considered.

Ecological validity for informing guidelines regarding physical activity prior to RMR testing should also be considered. Kuikman and colleagues conducted their study under highly rigorous and tightly controlled laboratory conditions (Kuikman et al. 2024). While this approach provides clear evidence on the effect of moderate intensity exercise on next‐day RMR (Kuikman et al. 2024), prescribing such specific intensities in real‐world free‐living settings can pose difficulties where athletes may not know their VO_2_max and/or train outside with variations in terrain and environment. Rating of perceived exertion (RPE) is a widely known subjective scale used to estimate the intensity of exercise based on how hard a person feels they are working (Garber et al. 2011). A variety of RPE scales have been shown to align well with key physiological markers such as ventilatory thresholds, blood lactate concentrations, and VO_2_ max in sedentary and active individuals including elite level athletes (Seiler and Kjerland 2006; Abe et al. 2015; Faulkner et al. 2007; Andrade et al. 2020; Scherr et al. 2013). Many athletes are familiar with how to interpret and apply RPE, both in reflecting on past sessions and in guiding future training. Even for those unfamiliar, a brief demonstration is usually sufficient to explain its use. Therefore, using RPE to guide intensity may represent a promising approach for informing recommendations on exercise in the free‐living environment prior to next‐day RMR testing. To the best of our knowledge, this has not been investigated.

Extraneous factors that may explain the impact of exercise on next‐day RMR are also important to consider. Exercise induced muscle damage, indicated by increases to creatine kinase (CK) blood levels and muscle soreness, has been shown to correspond with elevated RMR (Dolezal et al. 2000; Burt et al. 2014). Elevated CK has been observed post‐resistance exercise (Dolezal et al. 2000; Burt et al. 2014; Vieira et al. 2022), post match in several team sports (Aben et al. 2022; Hagstrom and Shorter 2018; Barth et al. 2019; Abián et al. 2016; Silva et al. 2018), and after various endurance races (Kanter et al. 1988; Magrini et al. 2017; Noakes et al. 1983; Hebisz et al. 2022; Belli et al. 2017). Urea is often measured in conjunction with CK as an indirect measure of muscle recovery by reflecting protein breakdown (Barth et al. 2019; Hartmann and Mester 2000; Hecksteden et al. 2017). For example, higher CK and urea levels have been shown when athletes were not recovered (after four consecutive training days) versus when they were recovered after a rest day (Barth et al. 2019). Urea has also been identified as a promising marker of recovery for endurance athletes (Hecksteden et al. 2016). These markers may therefore be useful to characterise the recovery status of the athlete, and whether this affects RMR.

The present study aims to determine (i) the impact of habitual endurance exercise, prescribed by RPE, on next‐day RMR and (ii) associations with other markers of exercise recovery, in male endurance athletes. It was hypothesised that self‐regulated moderate intensity endurance exercise in male athletes will not statistically significantly affect next‐day RMR when compared to rest, and that any differences if observed in RMR would be associated with changes to markers of exercise recovery. This knowledge will assist in informing future recommendations for exercise in the free‐living environment prior to next‐day RMR testing in athletes.

## 2 Methods

### 2.1 Study Design

This study adopted a randomised crossover design, comprising two testing sessions 7 days apart. Participants alternated between engaging in habitual exercise on the day prior to one session and resting on the day prior to the other session. The order was randomised using the online tool randomizer.org (Research Randomizer 2024). Measurements taken during both sessions included RMR, blood urea concentrations, and subjective measures of wellness via questionnaires. In addition, body composition was assessed at the first test session only. All assessments were conducted from 8:00 AM to 10:00 AM Monday to Friday. Participants were instructed to arrive to the laboratory via car. Participants were asked to avoid alcohol 24 h prior, and caffeine, nicotine, food and any drinks other than water from 12 h prior to their testing session. All testing took place at the Institute of Sport and Health at University College Dublin (UCD) from January 2022 to June 2022. Ethical approval was granted by the UCD Ethics Committee on the 14th January 2022 (Research Ethics Reference Number: LS‐22‐06‐Horner).

### 2.2 Participants

Inclusion criteria required athletes to be male, participating in either running, cycling, triathlon or rowing for the previous 6 months; be training at least 4 times a week for at least 1 h per session; have competed in their sport within the previous 6 months and have plans to compete in the following 6 months; be between the ages of 18 and 40 years old; have no known medical condition and must have performed a performance test in the previous 6 months (e.g. functional threshold power [FTP], 5‐km run time and 2‐km row time) and be able to self‐report this value. In the case where athletes may have completed more than one of these performance tests (e.g. triathletes), the performance test relating to the modality they used for their habitual exercise session was recorded. Exclusion criteria included any recent surgery, injury or medical condition or use of medications known to affect metabolic rate. Athletes were recruited through local athletic clubs and university sports teams, with the study details circulated via email and posted on club notice boards. Prior to participation, all individuals underwent a preliminary screening to assess eligibility based on the study criteria. Written informed consent was then obtained from eligible participants.

### 2.3 Testing Procedures

#### 2.3.1 Anthropometry and Body Composition

Height was measured in duplicate to the nearest 0.1 cm using a free‐standing stadiometer (seca, Hamburg, Germany). Body composition was assessed via air displacement plethysmography using the BODPOD (Cosmed, Rome, Italy).

#### 2.3.2 Resting Metabolic Rate

RMR was measured using the Q‐NRG (Cosmed, Rome, Italy) indirect calorimeter and a ventilated hood. Subjects removed their shoes and laid on a bed in the supine position. The lights were dimmed, and room temperature was kept at a constant range of 20°C–22°C. The test took 30 min to complete with the first 10 min being discarded. Gas exchange data were averaged over 30‐s intervals without additional filtering. RMR was calculated using the Weir equation (Weir 1949). From the final 20 min of data collection, the 10‐min segment with the lowest CV for RMR was identified, and the mean RMR across this 10‐min segment was used in the final analysis. To further characterise data stability, CV% was also calculated and reported for VO_2_, VCO_2_ and respiratory exchange ratio (RER) over the selected 10‐min steady‐state segment (see Table 1).

**TABLE 1:** Individual participant results for RMR, urea and questionnaire responses.

	Rested							Exercised							Rested–exercised
Participant number	RMR	RMR CV%	VO_2_ CV%	VCO_2_ CV%	RER CV%	Urea mg/dL	Wellness Q (total)	RMR	RMR CV%	VO_2_ CV%	VCO_2_ CV%	RER CV%	Urea mg/dL	Wellness Q (total)	RMR inter‐day CV%
1	2198	4.9	4.8	6.1	5.0	48.9	n/a	2025	3.9	3.7	5.0	3.1	49.3	n/a	4.1
2	1836	3.4	3.3	4.2	2.2	36.9	n/a	1943	1.4	1.5	1.9	2.0	52.9	n/a	2.8
3	2004	4.9	4.7	5.5	1.6	40.9	n/a	2130	6.6	6.9	8.1	4.7	40.3	n/a	3.0
4	1659	5.7	5.7	7.6	1.4	20	20	1720	6.2	6.0	8.5	6.4	49.1	13	1.8
5	2286	4.9	5.0	6.6	3.0	24.3	20	2218	5.4	5.6	8.1	2.1	35.2	16	1.5
6	2002	6.5	6.5	8.6	1.7	47.1	21	2647	4.4	4.7	5.6	4.1	49.6	14	13.9
7	2349	6.7	6.9	8.8	1.9	34.1	20	2156	4.4	4.2	5.2	2.8	30.7	19	4.3
8	1808	4.8	4.7	6.0	1.6	38.3	18	1559	4.1	3.8	4.4	2.2	38.3	18	7.4
9	1892	5.8	5.7	7.2	2.6	61.4	17	1922	3.4	3.4	4.6	1.5	42.3	15	0.8
10	1862	7.2	6.8	8.4	2.3	37.1	19	1731	5.0	4.8	5.5	3.1	34.3	19	3.6
11	1493	1.3	1.4	2.1	1.5	38.3	16	1637	1.1	1.3	2.2	3.7	42.9	16	4.6
12	2287	3.1	2.9	3.8	1.6	44.7	18	2240	5.4	5.5	7.4	2.3	38.8	15	1.0
13	1778	7.6	7.6	9.1	2.5	38.5	19	1800	5.8	5.4	6.5	3.2	40.5	20	0.6
Average	1958	4.9	4.9	6.2	2.2	39.3	18.8	1979	4.3	4.3	5.5	3.2	41.9	16.5	3.8
SD	251	1.6	1.6	2.0	1.0	10.0	1.5	289	1.6	1.6	2.1	1.3	6.5	2.3	3.4
Max	2349	7.2	6.9	8.8	5.0	61.4	21	2647	6.6	6.9	8.5	6.4	52.9	20	13.9
Min	1493	1.3	1.4	2.1	1.4	20	16	1559	1.1	1.3	1.9	1.5	30.7	13	0.6

Abbreviations: Average = column mean average; max = column maximum; min = column minimum; RER CV% = coefficient of variation for the respiratory exchange ratio values during the 10‐min steady‐state period; RMR = resting metabolic rate; RMR CV% = coefficient of variation for RMR values during the 10‐min steady‐state period; SD = column standard deviation; VCO_2_ CV% = coefficient of variation for carbon dioxide production values during the 10‐min steady‐state period; VO_2_ CV% = coefficient of variation for oxygen consumption values during the 10‐min steady‐state period; Wellness Q = wellness questionnaire by McLean et al. (2010).

#### 2.3.3 Reliability of RMR Measurement

Unpublished data from our laboratory (*n* = 30) indicate that the Q‐NRG indirect calorimeter has excellent inter‐day reliability with a typical error of measurement for RMR of 50 kcal/day (95% CI: 37 to 64 kcal/day), and a mean within‐subject coefficient of variation of 2.4% ± 1.6% (range 0.2%–6.0% and SEM = 0.29%; O'Neill and Horner 2020). In the present study, if participants’ inter‐day CV fell outside this range (inter‐day CV%: > 6%), their activities in the preceding 72 h were further characterised for reasons that may explain a greater between day variability in their RMR results.

#### 2.3.4 Urea

A finger prick blood sample (30 µL) was collected in a capillary blood‐collection tube. The blood sample was transferred onto a urea testing strip and placed in a reflectance photometry machine (Reflotron Plus, Woodley Scientific, Horwich, United Kingdom) for analysis. Normal ranges for urea activity using this method are 10–50 mgdL (Hartmann and Mester 2000).

#### 2.3.5 Subjective Measures of Recovery Via a Wellness Questionnaire

The wellness questionnaire by McLean and colleagues (McLean et al. 2010) was administered to assess different aspects of recovery including fatigue, muscle soreness, sleep quality, stress and mood.

#### 2.3.6 Habitual Exercise and Rest Conditions

For the habitual exercise condition, participants were instructed to perform an exercise session they were familiar with at an RPE of 3 or 4 on the modified CR‐10 scale (Foster et al. 2001). During the initial screening call, participants provided a detailed description of their training week, the days that they trained on, the composition of the training sessions and an estimated RPE rating for each session. To minimise interference with habitual training schedules, participants were booked in to complete their RMR measurement the day after they planned to complete a training session expected to be at an RPE of 3 or 4. Athletes were provided with a heart rate monitor to wear during exercise (Wahoo Tickr X [Wahoo Fitness, Atlanta, USA]). These data were recorded and analysed by the investigators. Age predicted maximum heart rate was calculated for each participant with the Tanaka et al. equation (HR_max_ = 208–0.7 × age (*y*)) (Tanaka et al. 2001).

For the rest condition, participants were instructed to abstain from all forms of physical activity (except those necessary for normal activities of daily living e.g. attending university) the day before the RMR measurement.

### 2.4 Statistical Analyses

Sample size calculations were conducted in G*Power (version 3.1). The study was powered to detect an approximate 3%–4% change in RMR (equating to 63 kcal for 13 participants), based on a within participant SD of 71 kcal observed in our unpublished data of 30 athletes, at 80% power and significance at *p* < 0.05. Fifteen participants were recruited to allow for ∼20% dropout. The Shapiro–Wilk test was used to assess normality. Comparison of RMR, urea, questionnaire responses and RER were made between conditions using paired *t*‐tests and Wilcoxon matched pairs signed rank tests for normally and nonnormally distributed data, respectively. Effect sizes (Cohen's d) were calculated for the differences in RMR, wellness scores, urea and RER between the exercise and rest conditions. In accordance with standard guidelines, effect sizes (ES) of approximately 0.2, 0.5 and 0.8 were interpreted as small, medium and large, respectively (Cohen 2013). Intra‐individual coefficients of variation (CV) were calculated for all variables between conditions. Spearman's rank correlation coefficients were used to assess relationships between markers of recovery and RMR. Statistical significance was accepted as *p* < 0.05. All data were analysed using the Statistical Package for the Social Sciences (SPSS, Version 21.0; SPSS Inc., Chicago, IL) and GraphPad Prism Version 9.0 for Windows (GraphPad Software, San Diego, CA).

## 3 Results

Fifteen male endurance athletes were recruited. Two were lost to follow up for the following reasons: illness (*n* = 1) and stopped responding to emails (*n* = 1). Thirteen male endurance athletes (cyclists *n* = 7, triathletes *n* = 5 and rower *n* = 1) were included in the final analysis (see Table 2 for participant characteristics).

**TABLE 2:** Subject characteristics presented as mean ± SD and range.

Characteristic	Mean ± SD	Range
Age (years)	25.4 ± 5.8	19–33
Height (m)	1.81 ± 0.09	1.68–2.03
Body mass (kg)	76.7 ± 12.8	63.2–113.7
BMI (kg/m^2^)	23.3 ± 2.1	19.3–27.7
Fat mass (kg)	12.4 ± 5.6	6.9–27.2
Fat mass (%)	15.9 ± 4.8	9.0–18.0
Fat‐free mass (kg)	63.6 ± 8.7	53.6–86.5
Fat‐free mass (%)	84.1 ± 4.8	76.1–91.0
FFMI (kg/m^2^)	19.34 ± 1.60	16.4–22.0
2 km erg time (*n* = 1) (mm:ss)	06:29	n/a
5 km time (*n* = 3) (mm:ss)	20:18 ± 00:51	19:37–21:31
FTP (*n* = 9) (watts/kg)	4.2 ± 0.5	3.5–5.3
Competitive status (national:recreational)	12:1	n/a

Abbreviations: BMI = body mass index; erg = rowing ergometer; FFMI = fat‐free mass index; FTP = functional threshold power, mm:ss.

### 3.1 Habitual Exercise Session

The mode of exercise performed by participants included indoor cycle on ergometer (*n* = 5), outdoor cycle (*n* = 4), outdoor run (*n* = 3) and indoor row on ergometer (*n* = 1). Exercise session duration was 111 ± 71 min (range: 57–327). Regarding the intensity of the exercise session, the mean heart rate was 128 ± 16 bpm (range: 104–167), percentage of maximum heart rate was 67.1% ± 8.2% (range: 53.4%–85.8%) and RPE was 3.31 ± 0.46 (range: 3–4; see Table 3).

**TABLE 3:** Habitual exercise session durations, heart rates, RPEs and modalities for individual participants.

Participant number	Session duration per HR monitor (minutes)	Session RPE	Session description (as described by participant)^a^	Average HR (bpm)	HR SD (bpm)	% of HR max (%)
1	67	3	Steady‐state rowing	127	25	66
2	116	3	Steady‐state easy cycle	124	29	64
3	123	3	Steady‐state easy cycle	117	21	60
4	327	4	Steady‐state easy cycle	120	20	62
5	156	3	Easy run (outside)	167	19	86
6	96	3	Steady‐state easy cycle	104	18	53
7	56	3	Easy run (outside)	132	22	69
8	83	4	Steady‐state easy cycle (outside)	138	22	74
9	64	3	Steady‐state easy cycle (outside)	130	10	69
10	74	3	Steady‐state easy cycle (outside)	144	22	78
11	158	3	Steady‐state easy cycle (outside)	119	13	64
12	62	4	Easy run (outside)	108	20	58
13	65	4	Steady‐state easy cycle	128	12	69

Abbreviations: bpm = beats per minute; HR = heart rate; HR max = calculated by 208–0.7 × age (*y*); RPE = rate of perceived exertion; SD = standard deviation.

^a^ Sessions were conducted indoors unless otherwise stated.

*Source:* Tanaka et al. (2001)

### 3.2 Resting Metabolic Rate

**FIGURE 1:**
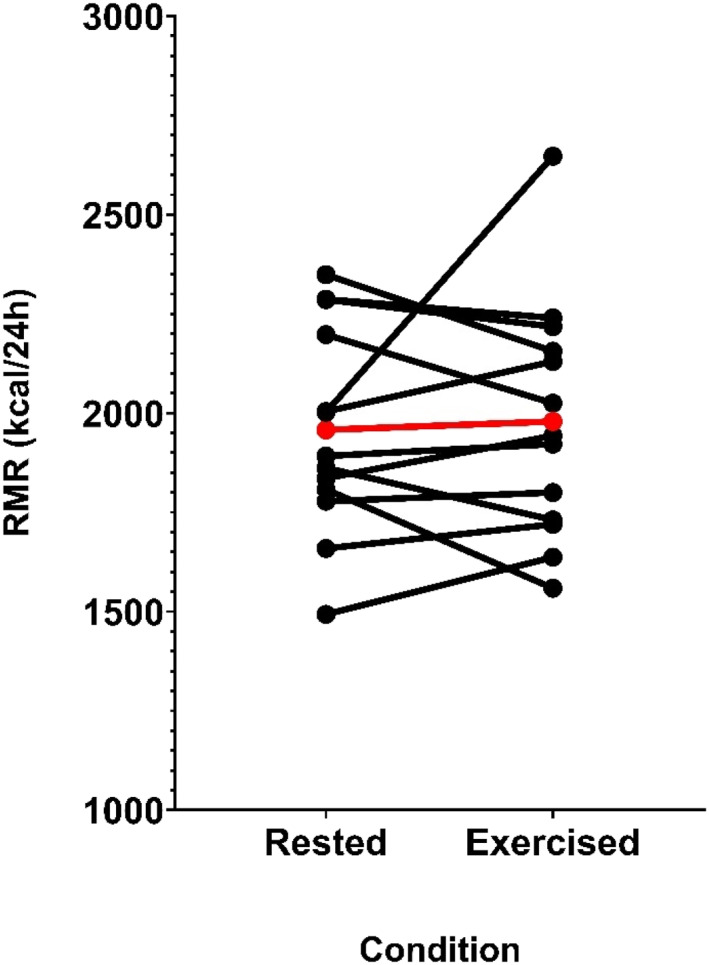
Within‐subject differences between rest and habitual exercise conditions for next‐day resting metabolic rate (RMR). Red line denotes group mean average, and each black line represents an individual participant.

There were no significant differences in RMR between rested and exercised conditions (1958 ± 251 vs. 1979 ± 289 kcal/day, mean Δ = 21 ± 227 kcal/day [95% CI = −116 to 158 kcal/day], *p* = 0.74 and ES = 0.09, Figure 1), or RER (0.78 ± 0.04 vs. 0.77 ± 0.08, mean Δ = 0.008 ± 0.054 [95% CI = −0.041 to 0.025 kcal/day] *p* = 0.62 and ES = −0.14). Inter‐day intra‐individual CVs for RMR and RER were 3.8% ± 3.4% (range: 0.6%–13.9%) and 2.9% ± 1.9% (range: 0.6%–6.3%), respectively. Regarding the stability of measurement, CVs for the 10‐min steady‐state periods were 5.1% ± 1.7% (range: 1.3%–7.6%) and 4.4% ± 1.6% (range: 1.1%–6.6%) for the rested and exercised conditions, respectively (see Table 1).

### 3.3 Blood Urea Levels

There were no significant differences in urea levels between rested and exercised conditions (39 ± 10 vs. 42 ± 6 mg/dL, mean Δ = 2.6 ± 11.5 kcal/day [95% CI = −4.4 to 9.5 kcal/day], *p* = 0.43 and ES = 0.23). Inter‐day intra‐individual CV for urea was 9.6% ± 11.5% (range: 0.0%–42.0%). The individual with the greatest difference in RMR between conditions (645 kcal, CV = 13.9%) had a urea inter‐day CV of 2.6%. Conversely, for the individual with the greatest urea inter‐day CV between conditions (42%), their inter‐day CV for RMR was 1.8% (61 kcal).

### 3.4 Subjective Markers of Recovery

Wellness questionnaire scores were analysed in *n* = 10 participants (missing data, *n* = 3 participants). Total scores for wellness were significantly higher in rested than exercise conditions (18.8 ± 1.6 vs. 16.5 ± 2.4, mean Δ = −2.3 ± 2.9 kcal/day [95% CI = −4.4 to −0.2 kcal/day], *p* = 0.03 and ES = −0.79). No statistically significant differences were observed for any subsections of the wellness questionnaire (fatigue: *p* = 0.25, muscle soreness: *p* = 0.13, sleep quality: *p* = 0.06, stress: *p* = 0.31 and mood: *p* = 0.99).

### 3.5 Correlates of RMR

No significant correlation was found between RMR differences and urea differences (*r* = 0.40 and *p* = 0.18) and between RMR differences and wellness score differences (*r* = −0.40, *p* = 0.25) (See Figure S1).

### 3.6 Individual Variability in Response

In two participants, both cyclists, an inter‐day CV of RMR > 6% was observed. This is outside the range of day‐to‐day variability in RMR previously described in our laboratory. One participant exhibited a pronounced increase in RMR (+645 kcal and CV = 13.9%) after their habitual exercise session, despite following the desired habitual exercise protocol similar to others (session duration: 96 min, average heart rate: 104 bpm and RPE: 3). However, the participant self‐reported that they were still experiencing muscle soreness after completing a national level cycling race 48 h prior. This was supported by a difference in their overall wellness score from 21 in the rest condition to 14 in the exercised condition and a difference in their specific score relating to muscle soreness from 4 (‘Feeling good’) to 2 (‘increase in soreness/tightness’). Conversely, another participant demonstrated a significant RMR increase (+249 kcal, inter‐day CV = 7.4%) following the rest condition. In anticipation for the scheduled rest day prior to their testing, the participant undertook a strenuous cycling session 48 h prior (90 km, > 60 min spent > 80% HR_max_). Despite this difference in RMR, their total score and subgroup scoring for the wellness questionnaire remained the same between conditions. Neither participant demonstrated elevated urea levels.

## 4 Discussion

The primary findings from the present study were (i) no statistically significant differences in next‐day RMR, between rest and habitual exercise conditions and (ii) no statistically significant associations between differences in RMR and markers of exercise recovery.

These findings contrast with some prior research reporting aerobic exercise elevates next‐day RMR (Maehlum et al. 1986; Hunter et al. 2006; Jamurtas et al. 2004). However, the exercise in these studies was conducted at high intensities (at least 70% VO_2_max) in participants who were classified as not participating in competitive sports (Maehlum et al. 1986), were overweight and previously sedentary prior to study enrolment (Hunter et al. 2006) or did not have their training, competitive or athletic status reported (Jamurtas et al. 2004). This may explain the difference in findings to the present study. Our participants completed the habitual exercise sessions at an RPE of 3–4, an RPE that has been shown to coincide with the first ventilatory threshold (VT1) in a study of highly trained and competitive male cross‐country skiers (Seiler and Kjerland 2006).

The present findings support the recent findings of Kuikman et al. demonstrating moderate intensity exercise does not have a statistically significant effect on next‐day RMR in endurance athletes in a highly controlled laboratory setting. Our findings extend this evidence to the free‐living environment, showing that moderate intensity (guided by RPE) does not significantly impact next‐day RMR in athletes. Another research study investigating the effects of exercise on EPOC in moderately trained females further supports these findings (Sedlock 1991). Although this study showed a significant increase in EPOC after participants cycled at 40% and 60% of VO_2_max, only 7–9 additional kilocalories were expended over 18–27 min. Overall, the evidence suggests that it is important to take into consideration the training status of the individual when considering the effects of aerobic exercise on RMR and indicates that moderate intensity habitual exercise does not significantly impact next‐day RMR in trained individuals.

Despite no statistically significant mean differences between conditions, two participants inter‐day CVs for RMR exceeded 6% (the maximum inter‐day CV we previously observed in athletes when instructed to rest on the day prior to repeat measurements). Interestingly, one participant's RMR was higher after the exercise condition and the other participant’s RMR was higher after rest. In the participant who experienced elevated RMR post‐exercise, his habitual exercise session characteristics were lower than average for duration and intensity (duration, 96 min and intensity, 52% HRmax, RPE 3) suggesting this did not impact the result. Taken together, these findings support the conclusion that habitual moderate intensity exercise may not significantly impact next‐day RMR and the observed variability in these participants is likely due to other reasons. However, both participants performed high‐intensity aerobic exercise within 48 h of the RMR measurement, which demonstrated elevated values. The physiological impact of high‐intensity aerobic activities, akin to those experienced in competitive racing, may extend elevation of metabolic rate beyond the 24‐h post‐exercise window typically reported in literature (Treuth et al. 1996; Maehlum et al. 1986). High‐intensity aerobic exercise may potentially align with the prolonged metabolic impact observed in intensive resistance training where elevations in RMR are observed up to 48 h (Dolezal et al. 2000). It may also impact the energetic status/energy availability of the participant prior to testing, which in turn could impact results (Siedler et al. 2023). Further research is required to investigate the effects of high‐intensity aerobic exercise and the impact of energy deficit within 48 h of RMR measurement.

Muscle soreness and urea levels did not correlate with RMR changes. The individual with the greatest change in RMR in our study had minimal changes in urea, and the individual with the greatest change in urea had minimal changes to RMR. Although urea is proposed as a promising marker of recovery for endurance athletes, it is emphasised that individual reference ranges need to be created to be used effectively (Barth et al. 2019; Hecksteden et al. 2017, 2016). Despite this, a possible explanation for our nonsignificant finding is that the habitual exercise session was not intense enough to elicit a significant impact on urea and thus increase RMR. This would require future research to develop individual reference ranges for urea for participants or incorporate athletes who already have individualised reference ranges established.

The wellness questionnaire outcomes pointed to notable variances in overall wellness scores, with wellness significantly reduced. Although, there was no association between wellness and RMR, the participant with the biggest difference in RMR reported increased muscle soreness. Markers of muscle damage and soreness have been shown to correspond with RMR in recovery (Dolezal et al. 2000). Therefore, monitoring muscle soreness may be useful to monitor when assessing RMR in endurance athletes, as a potential factor that may impact results.

Some methodological aspects of the study should be considered. This study’s scope was confined to male endurance athletes, with a specific focus on moderate intensity exercise conducted within 24 h prior to RMR measurement. Further research is required to examine the potential influence of high‐intensity activities performed up to 48 h before testing. Previous research on the effects of moderate intensity exercise on EPOC in moderately trained females (Sedlock 1991) implies our findings could be expected in females also. Nonetheless, further studies should include female endurance athletes. In addition, the impact of other modalities of exercise including concurrent training warrant further research.

The only instruction to athletes regarding the habitual exercise sessions was to perform the session at an RPE of 3–4. Compared to a laboratory‐based study, this enhances the ecological validity of the findings, making them relevant for future research and practical applications in measuring RMR. Although heart rate was measured and characteristics of the session were recorded, other factors such as power output, blood lactate and geographical factors such as elevation change during outdoor sessions were not recorded. Future studies could adopt a similar approach but would benefit further by including these measures. In addition, the inclusion of other markers of recovery such as CK would be beneficial (Dolezal et al. 2000; Burt et al. 2014; Vieira et al. 2022; Aben et al. 2022; Hagstrom and Shorter 2018; Barth et al. 2019; Abián et al. 2016; Silva et al. 2018; Kanter et al. 1988; Magrini et al. 2017; Noakes et al. 1983; Hebisz et al. 2022; Belli et al. 2017). This was not possible in the present study due to logistical constraints. Finally, our study cannot confirm a strict judgement of no difference between the tested conditions, as formal equivalence testing was not conducted and further studies with larger sample sizes are needed for this. Nevertheless, our findings provide valuable preliminary evidence that can be incorporated into future systematic reviews and meta‐analyses to help clarify these findings more definitively.

In conclusion, our data indicate no statistically significant difference in next‐day RMR between self‐regulated moderate intensity exercise and rest conditions, suggesting habitual moderate intensity exercise may be feasible on the day prior to testing. However, further studies of larger sample sizes and including females are required before an amendment could be considered to the upper limit of current best practice guidelines (Fullmer et al. 2015), particularly for endurance athletes. Such a revision could offer more adaptable scheduling opportunities for testing athletes in both research and practical applications. Caution is still warranted for high‐intensity exercise within 48 h of testing as this also requires further study.

## Conflicts of Interest

The authors declare no conflicts of interest.

## Supporting information

Figure S1
