# Supplementary material for: Self‐Regulated Moderate Intensity Habitual Exercise and Next‐Day Resting Metabolic Rate in Male Endurance Athletes: Implications for Athlete Testing
Source: Eur J Sport Sci. 2025 Jul 10;25(7):e70011. doi: 10.1002/ejsc.70011 (PMC12244387; doi:10.1002/ejsc.70011)
Supplement: Supplementary file 1 — Figure S1 [file EJSC-25-e70011-s001.docx]

**Supplementary Materials**

**
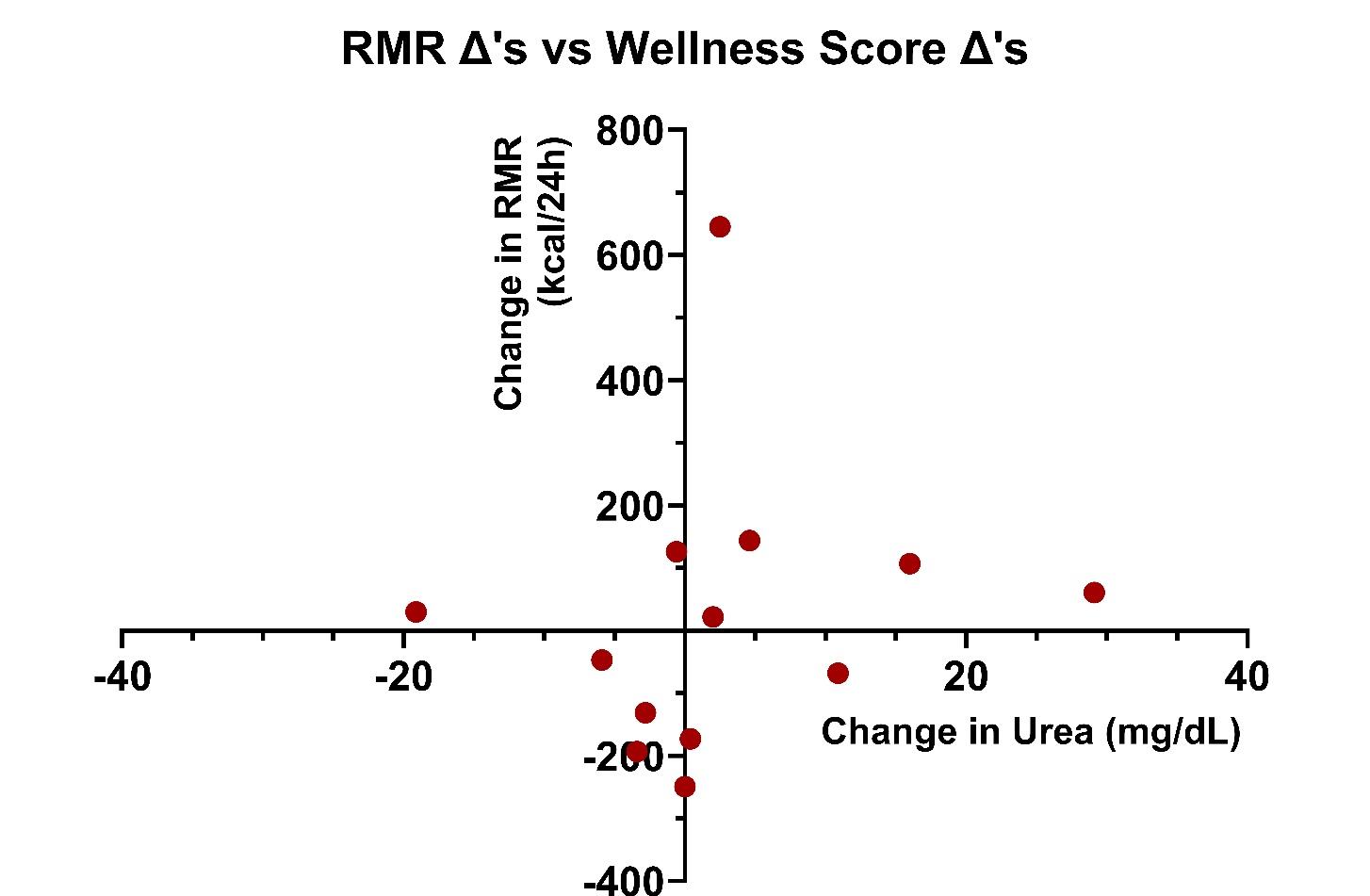
**


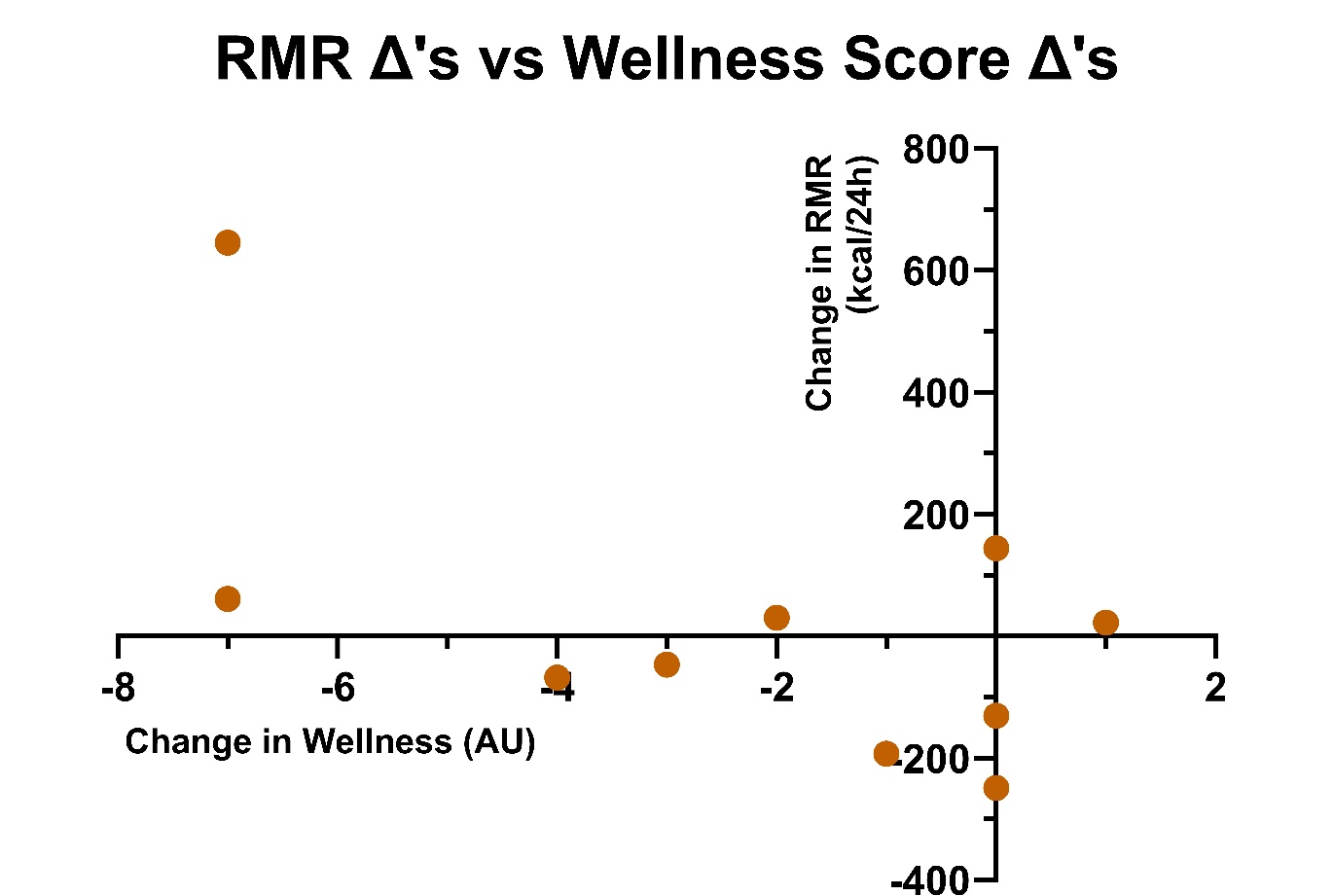


***Figure 1: Spearman rank correlation analysis between RMR differences and urea differences (top) and between RMR differences and wellness score differences (bottom). Differences calculated as exercise-rested values.***
